# Natural 2D layered mineral cannizzarite with anisotropic optical responses

**DOI:** 10.1038/s41598-022-14046-8

**Published:** 2022-06-15

**Authors:** Arindam Dasgupta, Xiaodong Yang, Jie Gao

**Affiliations:** 1 Department of Mechanical and Aerospace Engineering, Missouri University of Science and Technology, Rolla, MO 65409 USA; 2 Department of Mechanical Engineering, Stony Brook University, Stony Brook, NY 11794 USA

**Keywords:** Two-dimensional materials, Nonlinear optics

## Abstract

Cannizzarite is a naturally occurring mineral formed by van der Waals (vdW) stacking of alternating layers of PbS-like and Bi_2_S_3_-like two-dimensional (2D) materials. Although the PbS-type and Bi_2_S_3_-type 2D material layers are structurally isotropic individually, the forced commensuration between these two types of layers while forming the heterostructure of cannizzarite induces strong structural anisotropy. Here we demonstrate the mechanical exfoliation of natural cannizzarite mineral to obtain thin vdW heterostructures of PbS-type and Bi_2_S_3_-type atomic layers. The structural anisotropy induced anisotropic optical properties of thin cannizzarite flakes are explored through angle-resolved polarized Raman scattering, linear dichroism, and polarization-dependent anisotropic third-harmonic generation. Our study establishes cannizzarite as a new natural vdW heterostructure-based 2D material with highly anisotropic optical properties for realizing polarization-sensitive linear and nonlinear photonic devices for future on-chip optical computing and optical information processing.

## Introduction

In layered van der Waals (vdW) materials, individual layers of chemically bonded atoms are stacked and held by weak vdW interaction. Therefore, these materials can be easily thinned down to nanometer thicknesses, where the unique charge carrier confinement in the two-dimensional (2D) layers gives rise to extraordinary electronic and optical properties^1,2^. The discovery of graphene, hexagonal boron nitride, and transition metal chalcogenides has paved the way for utilization of layered vdW materials to develop various on-chip optical, electronic, and mechanical devices of molecular thicknesses^3–6^. Further, the physical properties of these materials are strongly dependent on their specific crystal structures. Hence, the structural anisotropy in layered vdW materials will enable the possibility of varying their physical properties when probed along different spatial directions, providing an extra degree of freedom for manipulating the responses of these materials under external stimuli. Therefore, black phosphorus (BP), ReS_2_, ReSe_2_, GeSe, and other group-IV monochalcogenides exhibiting in-plane anisotropy have been explored for achieving many new functionalities that are not possible with conventional isotropic vdW materials, for example, polarization-sensitive photodetectors^7–10^, modulators^11^, synaptic devices^12^, digital inverters^13^, and anisotropic resistors^14^, which can be used as building blocks for future on-chip photonic integrated circuits. Apart from the optoelectronic and linear optical properties, the nonlinear optical properties of layered vdW materials are also determined by the structural anisotropy in the crystal^15–18^. Therefore, anisotropy in the nonlinear optical properties of these materials can be exploited for realizing high-performance nonlinear optical devices for all-optical signal processing and optical communication.

The structural anisotropy in the crystal can also be induced by creating vdW heterostructures via stacking individual 2D material layers of different material types. The optical properties of vdW heterostructures can be easily tailored by the stacking symmetries and strategies of individual 2D material layers, which provides the opportunity of achieving diverse functionalities beyond the reach of each individual layer. Over the last decade, the fabrication of vdW heterostructures has advanced the reach of layered vdW materials to realize ultracompact photonic and optoelectronic devices covering a broad spectral range from visible to mid-IR for optical communication and sensing^19^, transistors^20,21^, photodetectors^22–24^ and ultrafast lasers^25^. Widely used methods for the fabrication of vdW heterostructures include layer-by-layer sequential chemical synthesis and mechanical restacking of individual materials^25–30^. However, these methods are technically challenging, time consuming, and susceptible to induce unwanted defects and interlayer adsorbates in the fabricated vdW heterostructures. Recently, mechanical exfoliation of natural vdW superlattices such as cylindrite^31,32^, franckeite^33,34^, and lengenbachite^35^ has come into foray as an alternative way to fabricate defect and adsorbate free vdW heterostructures. Cylindrite and franckeite belong to the material class where the superlattice is made of the alternative PbS-type and SnS_2_-type layers, while the superlattice of lengenbachite consists of the alternative PbS-type and As_2_S_3_-type layers. In these cases, the formed vdW heterostructures exhibit in-plane structural and optical anisotropy, even though the individual layers of PbS, SnS_2_ or As_2_S_3_ are structurally isotropic in nature. Recently, several reports on these materials have demonstrated their anisotropic optical behavior in the linear and nonlinear optical regime^32,35–37^. Therefore, it is an imperative to search for other materials which can be mechanically exfoliated to obtain ultrathin vdW heterostructures composed of other kinds of 2D material layers.

With that hindsight, here we introduce natural layered mineral cannizzarite as a new vdW heterostructure with anisotropic structural and optical properties. Cannizzarite is a rare lead bismuth sulfosalt mineral with the nominal composition of Pb_46_Bi_54_(S,Se)_127_, which is composed of the vdW stacking of PbS-like and Bi_2_S_3_-like 2D material layers. It was discovered in 1925 at the crater of La Fossa, Vulcano Island, Italy and named in honor of the Italian chemist Stanislao Cannizzaro^38^. It occurs as a sublimation product in the deepest part of the fumaroles at high temperature (550–615 °C). Cannizzarite forms aggregates of silvery-gray, thin-bladed tabular and leafy crystals with a Mohs scale hardness of 2, which are often warped and may have frayed ends^39–41^. Recently, the thermoelectric properties of Pb_5_Bi_6_Se_14_, a member of the cannizzarite homologous series with a general formula of [(PbSe)_5_]_m_[(Bi_2_Se_3_)_3_]_n_ with m = 1 and n = 1, has been investigated for high thermoelectric figure of merit as well as anisotropic electrical and thermal transport properties^42,43^. For one potential application, natural 2D layered mineral cannizzarite can be considered as high-performance thermoelectric material for the next-generation energy conversion. In the vdW heterostructure of cannizzarite, the PbS-type and Bi_2_S_3_-type layers are incommensurate to each other, so that the forced commensuration between these two lattices will give rise to in-plane structural anisotropy. Here we show that ultrathin vdW heterostructures of alternating PbS-type and Bi_2_S_3_-type 2D material layers can be achieved through the mechanical exfoliation of a natural cannizzarite rock. The in-plane structural and optical anisotropy of the exfoliated thin cannizzarite flakes are studied in detail. First, the anisotropic crystal structure, and the bulk and surface chemical composition of cannizzarite are determined by transmission electron microscopy (TEM), energy-dispersive X-ray spectroscopy (EDXS), and X-ray photoelectron spectroscopy (XPS) analysis. Furthermore, the in-plane structural anisotropy of cannizzarite flakes is probed by angle-resolved polarized Raman spectroscopy. The optical anisotropy induced linear dichroism is observed by performing polarization-dependent absorption measurements. Moreover, the effect of crystal structural anisotropy on the optical nonlinearity is investigated by measuring the polarization-dependent THG response in cannizzarite flakes, and by estimating the third-order nonlinear susceptibility. The demonstrated results represent cannizzarite as a promising candidate for expanding the current existing 2D material library of natural vdW heterostructures and can also be harnessed to realize ultracompact polarization-sensitive anisotropic photonic and optoelectronic devices for future on-chip optical computing and optical information processing.

## Results

### Crystal morphology and chemical composition of cannizzarite

**Figure 1:**
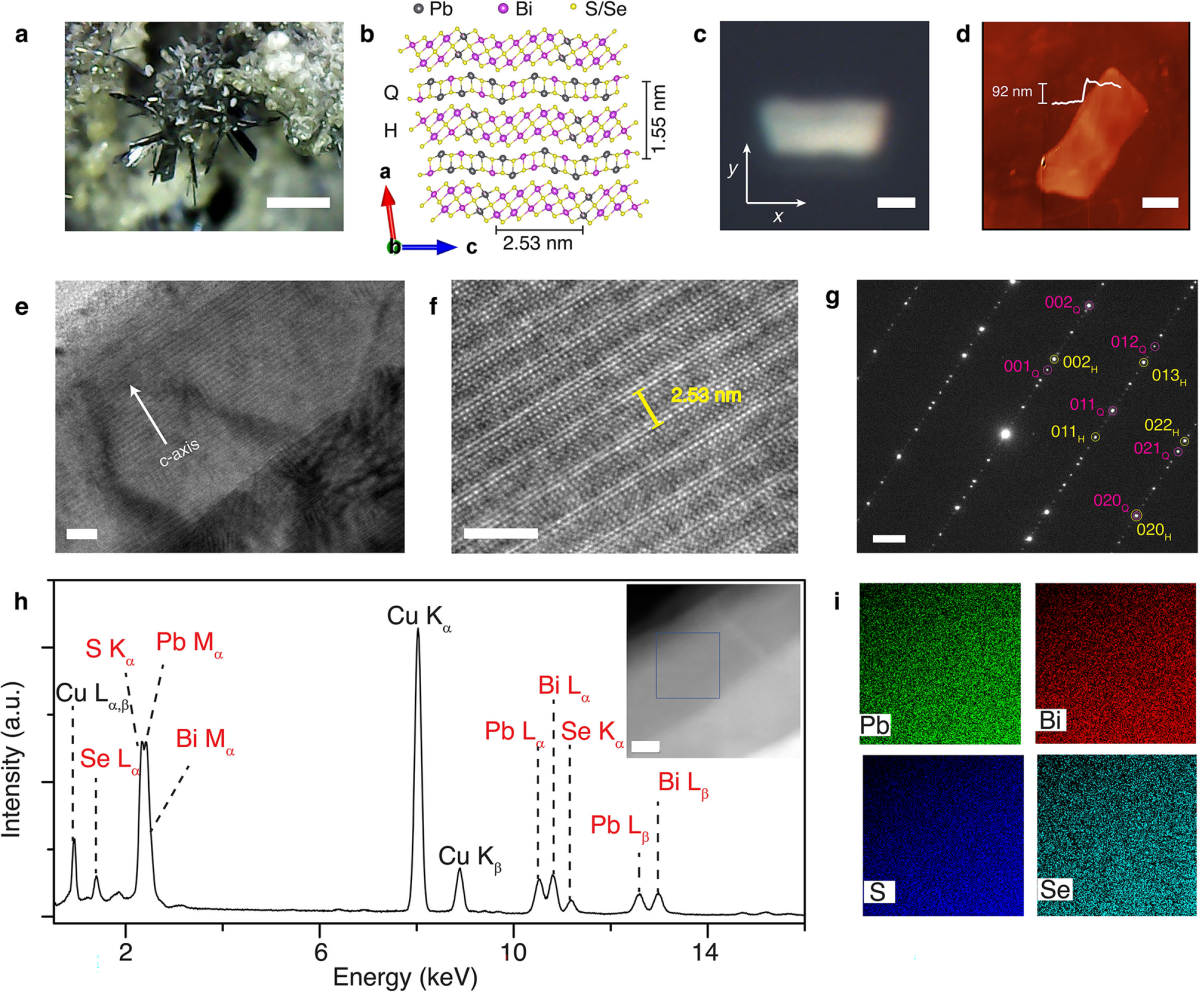
(**a**) Optical image of the cannizzarite mineral rock where clusters of thin tabular and leafy crystal blades with silvery-gray color are attached on gray perlitic andesite matrix. Scale bar is 2 mm. (**b**) Schematic diagram of the crystal structure of cannizzarite composed of alternating PbS-like pseudo-tetragonal layer (Q-layer) and Bi_2_S_3_-like pseudo-hexagonal layer (H-layer). (**c**) Reflection optical microscope image of an exfoliated cannizzarite flake. The *x*- and *y*-axes signify the reference axes. (**d**) Corresponding AFM image with the line profile in the inset shows that the flake is 92 nm thick. Scale bars are 2 μm. (**e**) Low-magnification TEM image of a mechanically exfoliated thin cannizzarite flake showing the characteristic interlayer moiré patterns along the *c*-axis of the crystal. Scale bar is 10 nm. (**f**) Zoomed-in HRTEM image showing the atomic arrangements. Scale bar is 5 nm. (**g**) Corresponding SAED pattern. Scale bar is 1 nm^−1^. (**h**) Averaged EDXS spectrum acquired from the highlighted region of the flake shown in the inset (scale bar is 10 nm), confirming the presence of all the main elements Pb, Bi, S, and Se. (**i**) Corresponding TEM-EDXS map of the main elements Pb, Bi, S, and Se.

Figure 1a is a picture of a typical naturally formed cannizzarite mineral rock from La Fossa Crater, Vulcano Island, Lipari, Eolie Islands, Messina Province, Sicily, Italy, where silvery-gray, thin-bladed tabular and leafy cannizzarite crystals are attached to gray perlitic andesite matrix. Figure 1b is a schematic showing the simplified crystal structure of cannizzarite, where the crystal is formed by the vdW stacking of the alternated layers of incommensurate lattices of two-atom thick pseudo-tetragonal (Q) layer of (Pb,Bi)(S,Se) and double-octahedral five-atom thick pseudo-hexagonal (H) layer of (Bi,Pb)_2_(S,Se)_3_. Previous studies indicate that cannizzarite exhibits a monoclinic crystal structure belonging to P2_1_/m space group^44,45^. The lattice constants of Q-layer are ${a}_{\mathrm{Q}}=15.48$ Å, ${b}_{\mathrm{Q}}=4.09$ Å, ${c}_{\mathrm{Q}}=4.13$ Å, and ${\beta }_{\mathrm{Q}} = 98.56^\circ$, whereas the lattice constants of the H-layer are ${a}_{\mathrm{H}}=15.46$ Å, ${b}_{\mathrm{H}}=4.09$ Å, ${c}_{\mathrm{H}}=7.03$ Å, ${\beta }_{\mathrm{H}} = 98.00^\circ$. The crystal elongation direction is along the *c*-axis and the layer stacking period is $15.5$ Å along the $a$-axis. The formula of cannizzarite Pb_46_Bi_54_(S,Se)_127_ consists of alternating Q-layer of [(Pb,Bi)_46_(S,Se)_46_] and H-layer of [(Pb,Bi)_54_(S,Se)_81_]. It is evident that the two constituent lattices are incommensurable along the *c-*axis, therefore according to the vernier principle, one super-cell of the cannizzarite crystal is defined by the long-range match between the *m* number of H-subcells and *n* number of Q subcells with $m{c}_{\mathrm{H}}=n{c}_{\mathrm{Q}}$. Previous studies on the material have indicated *m*H:*n*Q homologous members of the cannizzarite variable-fit series with 3H:5Q, 7H:12Q, 10H:17Q, 27H:46Q^46–48^. Other possible *m*H:*n*Q matches have also been predicted for the cannizzarite series, where the change in the number of *m*Q:*n*H means a variation in the Pb:Bi ratio and cation:anion ratio in the crystal^46–48^. Since the constituent H-layer and Q-layer are periodically stacked and held by weak vdW interaction, cannizzarite can be mechanically exfoliated. The bulk cannizzarite minerals are exfoliated using Nitto tape (SPV 224) to obtain thin flakes with different thicknesses on quartz substrate. Figure 1c is the reflection optical microscope image of one exfoliated cannizzarite flake. The surface topography of the crystal is analyzed using atomic force microscopy (AFM). The height profile of the flake in the AFM image in Fig. 1d shows that the thickness of the flake is 92 nm approximately.

First, the crystal structure of mechanically exfoliated cannizzarite flakes is characterized by TEM analysis, where the exfoliated flakes are transferred onto a TEM copper grid. Figure 1e is the TEM image of a cannizzarite flake where an in-plane striped pattern of periodic darker and lighter areas is clearly observed, indicating an out-of-plane rippling modulated perpendicular to the surface. These rippled structures known as interlayer moiré patterns are common in vdW heterostructures made by stacking incommensurate 2D material layers. In cannizzarite, the rippled structures are formed to force the commensuration between the incommensurate Q-type (Pb,Bi)(S,Se) lattice and H-type (Bi,Pb)_2_(S,Se)_3_ lattice. The constituent Q-type and H-type lattices are incommensurate along the *c-*axis of the crystal, which signifies the direction of rippling. The out-of-plane rippling also induces strong in-plane structural anisotropy. Similar interlayer moiré patterns have been observed in other natural vdW heterostructures of cylindrite^31,32^, franckeite^33,34,37^, and lengenbachite^35^. The zoomed-in high-resolution (HR) TEM image in Fig. 1f shows that the fringe period along the rippling direction of the *c-*axis is around 2.53 nm, which is approximately half of the lattice constant *c* = 4.95 nm of the supercell, suggesting that the subcell match for the current natural cannizzarite crystal is 7H:12Q. Figure 1g shows the selected area electron diffraction (SAED) pattern along the surface normal to the [100] crystal zone axis. The reflections corresponding to individual Q-type and H-type layers are clearly visible. The presence of many weak supperlattice spots along the [001] direction further confirms that the vdW heterostructure of cannizzarite is formed by the stacking of incommensurate Q-type and H-type layers. Furthermore, the chemical composition of the exfoliated cannizzarite flakes is analyzed by EDXS. Figure 1h plots an averaged EDXS spectrum acquired from the flake, which indicates the presence of all the main elements in Pb, Bi, S, and Se. The signal of Cu is present due to the copper TEM grid. The compositional stoichiometry of the cannizzarite flake is deduced by the quantification of each element in the crystal, as listed in Table 1. The approximate chemical formula is estimated as Pb_59_Bi_67_S_97_Se_30_. Although the approximate chemical formula is not charge balanced, it is close to that of the previously determined chemical formula of cannizzarite of Pb_46_Bi_54_(S,Se)_127_^44,45^. The over estimation of Pb and Bi atoms may be attributed to the low sensitivity of EDXS and the heavy overlap between S Kα, Pb Mα, and Bi Mα peaks. The EDXS mapping of the individual elements in Fig. 1i aquired from the highlighted area shown in the dark-field TEM image in the inset of Fig. 1h indicates that each element is uniformly distributed. A homogeneous distribution of all the elements in the crystal is a further indication of cannizzarite being a vdW heterostructure of (Pb,Bi)(S,Se) and (Bi,Pb)_2_(S,Se)_3_ individual layers.

**Table 1:** EDXS quantification of elements in cannizzarite.

Element	Average concentration (at%)
Pb	23.39 ± 2.36
Bi	26.34 ± 2.65
S	38.24 ± 1.25
Se	12.03 ± 0.41

**Figure 2:**
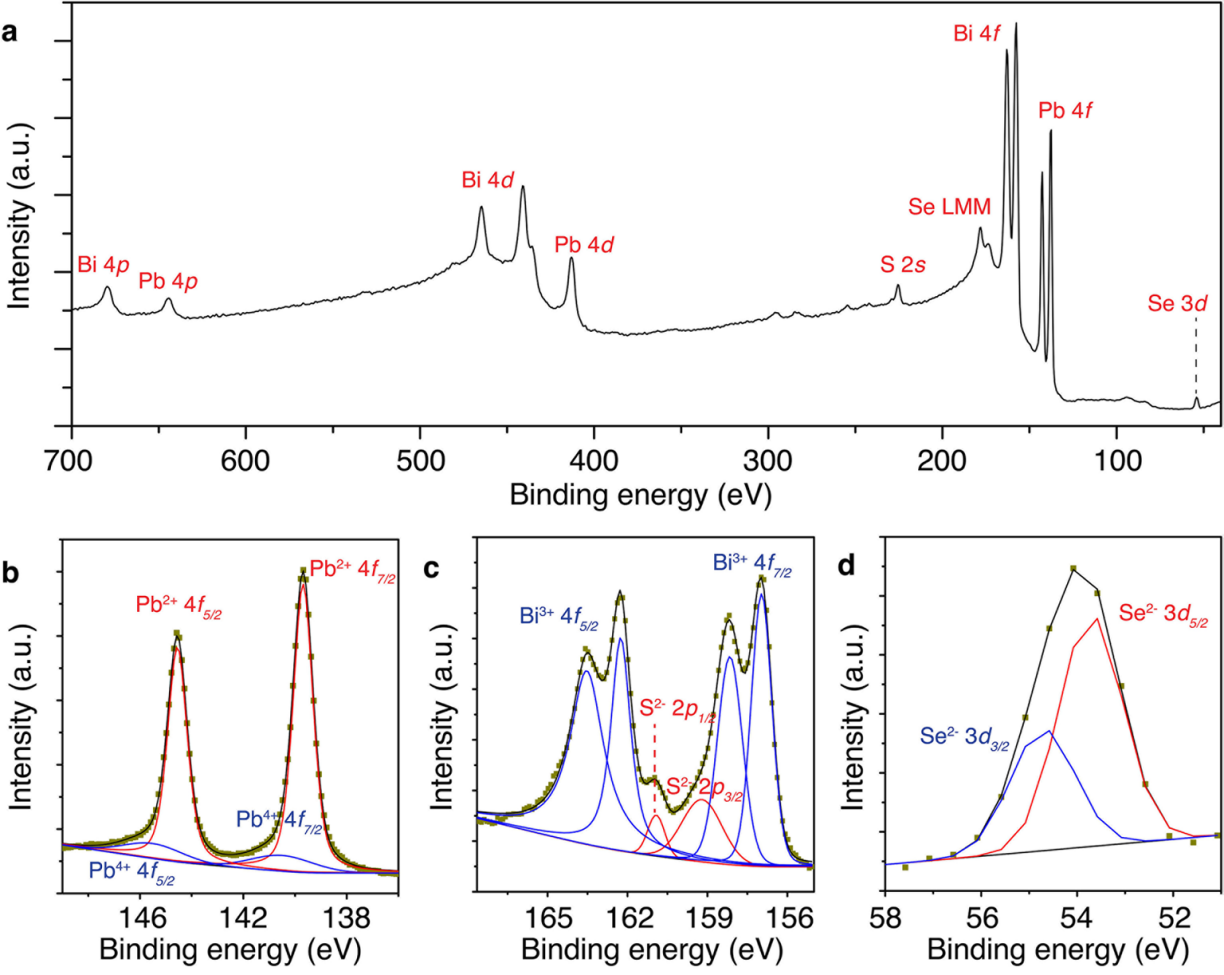
(**a**) Averaged XPS survey spectrum of cannizzarite obtained from multiple measurements. (**b**–**d**) High-resolution spectra around the binding energy regions of Pb 4f., Bi 4f., S 2*p*, and Se 3*d*, respectively.

The surface composition and stability of the air-aged exfoliated cannizzarite flakes are investigated through XPS measurement. Figure 2a shows the averaged XPS survey spectrum, which confirms the presence of all the major elements Pb, Bi, S, and Se. The fact that there is no oxygen peak in the XPS spectrum suggests that the exfoliated cannizzarite crystals are stable in ambient conditions. The compositional stoichiometry of the cannizzarite flake under study is shown in Table 2, giving the approximate surface chemical formula of cannizzarite as Pb_47_Bi_56_S_107_Se_20_. The deduced formula is very close to that of the previously reported charge-balanced formula of cannizzarite. A slight deviation from the actual formula might be due to the heavy superposition between the Bi 4f. and S 2*p* peaks. Further, the oxidation states of each element are determined from the XPS results. Figure 2b–d are the high-resolution spectra around Pb 4f., Bi 4f., S 2*p*, and Se 3*d* binding energy regions. Figure 2b suggests that Pb is present in the crystal at 2^+^ and 4^+^ states at 89% to 11% ratio. While Bi atoms are present at 3^+^ state according to Fig. 2c. S and Se are solely present at 2^−^ state as shown in Fig. 2c and d.

**Table 2:** XPS quantification of elements in cannizzarite.

Element	Average concentration (at%)
Pb	20.43 ± 1.39
Bi	24.34 ± 2.54
S	46.52 ± 2.27
Se	8.71 ± 1.02

### Determination of structural anisotropy in cannizzarite by Raman spectroscopy

**Figure 3:**
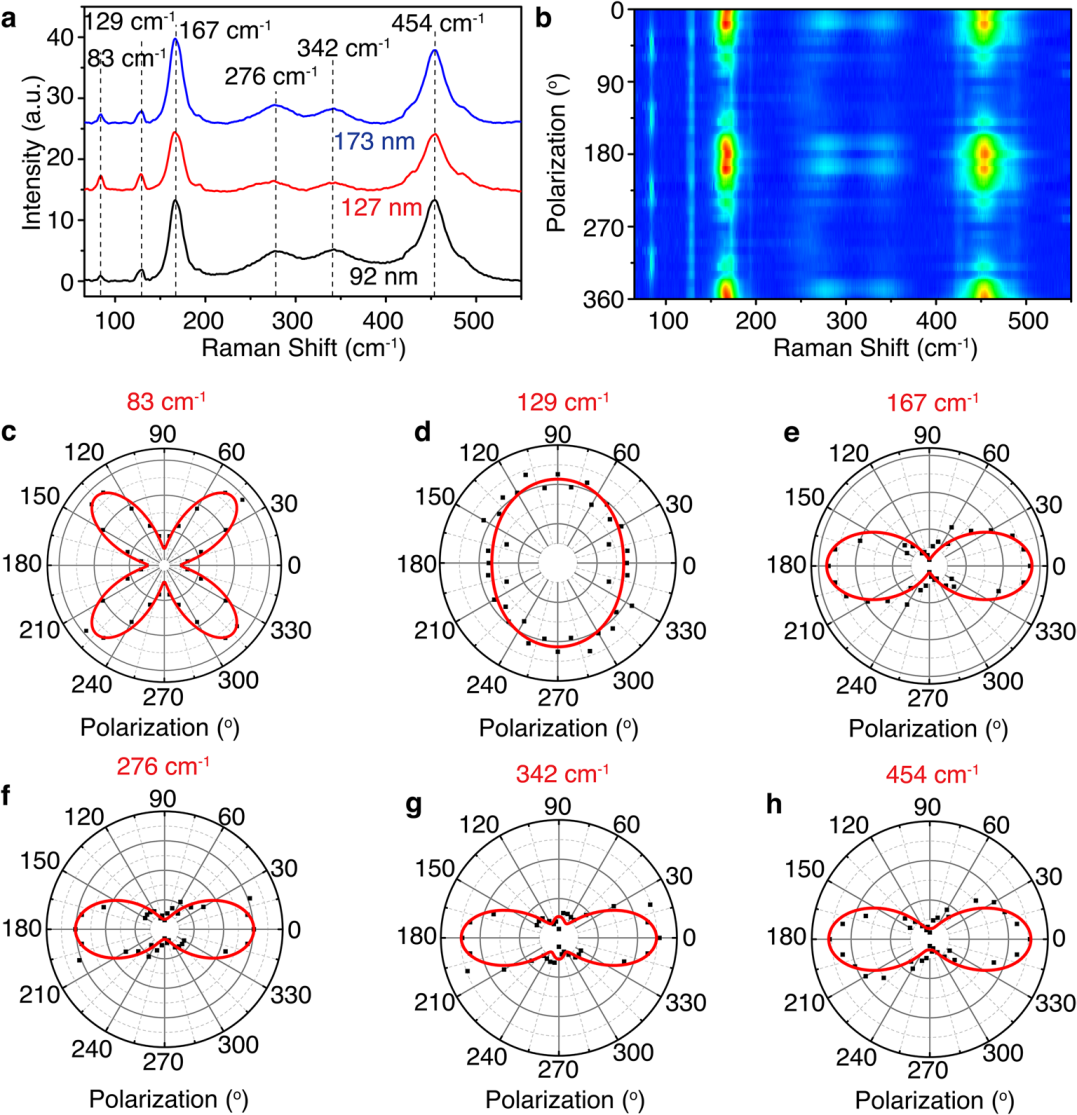
(**a**) Raman spectra acquired from three cannizzarite flakes with the thicknesses of 92, 127, and 173 nm. (**b**) Contour color map of the parallel polarization components of Raman spectra as a function of the incident linear polarization angle from the same 92 nm flake as shown in Fig. 1c. (**c**–**h**) Polar plots of the Raman intensity variations of the parallel polarization components for the Raman modes at 83, 129, 167, 276, 342, and 454 cm^−1^, respectively.

Figure 3a shows the Raman spectra acquired from three cannizzarite flakes with the thicknesses of 92, 127, and 173 nm by exciting the flakes with a 632.8 nm wavelength He–Ne laser. A series of different Raman peaks are observed within the 65–550 cm^−1^ frequency range at 83, 129, 167, 276, 342, and 454 cm^−1^. No obvious shift in the Raman peaks is observed depending on the thickness of the cannizzarite flake, due to the incommensurate vdW stacking of the alternating Q-type and H-type layers. The Raman modes of cannizzarite can be assigned based on the Raman spectra of the component binary sulfides of galena PbS^49,50^, bismuthinite Bi_2_S_3_^51,52^, clausthalite PbSe^53,54^, and guanajuatite Bi_2_Se_3_^55,56^. The 83 cm^−1^ peak is assigned as the B_1g_ mode of Bi_2_S_3_ (81 cm^−1^). The 129 cm^−1^ peak is related to the combination of longitudinal optical phonons of PbSe (138 cm^−1^) and the E_g_ mode of Bi_2_Se_3_ (133 cm^−1^). The peak at 167 cm^−1^ is attributed to the B_1g_ mode of Bi_2_S_3_ (168 cm^−1^) and the A_1g_ mode of Bi_2_Se_3_ (175 cm^−1^). The 276 cm^−1^ peak corresponds to the B_1g_ mode of Bi_2_S_3_ (277 cm^−1^) and the first overtone of the longitudinal optical phonons of PbSe (274 cm^−1^). The peak at 342 cm^−1^ is associated with the higher-order Raman scattering of PbSe (350 cm^−1^). The 454 cm^−1^ peak represents the first overtone of the longitudinal optical phonons of PbS (454 cm^−1^). The structural anisotropy in the monoclinic crystal of cannizzarite is further probed by the angle-resolved polarized Raman spectroscopy. Previously, angle-resolved polarized Raman spectroscopy has been employed to determine the structural anisotropy in monoclinic 2D crystals such as MoTe_2_^57^ and GeAs^18^. Moreover, the technique has also been used for probing the structural anisotropy in other natural vdW heterostructures such as cylindrite^32^, franckeite^36,37^, lengenbachite^35^, and nagyágite^58^. The contour color map in Fig. 3b shows the Raman intensity variation in the parallel polarization configuration as a function of the incident polarization angle ($\theta$) with respect to the *x*-axis ($\theta =0^\circ$). The flake used for this study is the same 92 nm flake shown in Fig. 1c. Both A_g_ and B_g_ Raman modes are present, which is expected in case of monoclinic crystals. It is evident that the intensities of the Raman modes vary periodically depending on the excitation polarization angle. By fitting such angle-dependent Raman intensities with the Raman tensor of the monoclinic crystal system of cannizzarite, the *c-*axis of the crystal can be determined. Considering that cannizzarite exhibits a monoclinic crystal structure belonging to P2_1_/m space group, and the $c, b$, and $a$-axes of the crystal are oriented along $x$-, $y$-, and $z$- directions, the Raman tensors of the A_g_ and the B_g_ mode can be written as,

1 $${{\varvec{\overset{\lower0.5em\hbox{$\smash{\scriptscriptstyle\leftrightarrow}$}} {R}}}}_{{A}_{g}}=\left(\begin{array}{ccc}c&\quad 0&\quad d\\ 0&\quad b&\quad 0\\ d&\quad 0&\quad a\end{array}\right)$$

2 $${{\varvec{\overset{\lower0.5em\hbox{$\smash{\scriptscriptstyle\leftrightarrow}$}} {R}}}}_{{B}_{g}}=\left(\begin{array}{ccc}0&\quad f&\quad 0\\ f&\quad 0&\quad g\\ 0&\quad g&\quad 0\end{array}\right)$$

where *a*, *b*, *c*, *d*, *f*, and *g* are the complex components of the Raman tensors. Mostly, these components are treated as real values since the imaginary part of the Raman susceptibility is negligible in transparent materials. However, for cannizzarite flakes with significant absorption, these components are complex elements, and can be represented as ${R}_{ij}=\left|{R}_{ij}\right|{e}^{i{\phi }_{{R}_{ij}}}$. Therefore, the Raman intensity for a particular incident polarization is calculated as $I={\left|{\widehat{e}}_{s}\cdot R\cdot {\widehat{e}}_{i}\right|}^{2}$, where the unit polarization of the incident beam is $${\widehat{e}}_{i}=\left[\begin{array}{ccc}\cos\theta & \sin\theta & 0\end{array}\right]$$, and the unit polarization of the scattered beam is expressed as $${\widehat{e}}_{s}=\left[\begin{array}{ccc}\cos\theta & \sin\theta & 0\end{array}\right]$$ for the parallel polarization. Here, $\theta =0^\circ$ corresponds to the incident linear polarization being along the $x$-direction. Therefore, the angle-dependent Raman intensities of the parallel polarization components of the A_g_ and B_g_ modes can be written as^58^,

3 $${I}_{\parallel }\left({A}_{\mathrm{g}}\right)\propto {\left|c\right|}^{2}{\cos}^{4}\theta +{\left|b\right|}^{2}{\sin}^{4}\theta +\frac{\left|c\right|\left|b\right|}{2}\cos{\phi }_{cb}{\sin}^{2}2\theta$$

4 $${I}_{\parallel }\left({B}_{\mathrm{g}}\right)\propto {\left|f\right|}^{2}{\sin}^{2}2\theta$$

where ${\phi }_{cb}$ is the phase difference between $c$ and $b$. From Eq. (3), it is evident that the variation of ${I}_{\parallel }\left({A}_{\mathrm{g}}\right)$ will exhibit a two-fold rotational symmetry, while ${I}_{\parallel }\left({A}_{\mathrm{g}}\right)$ reaches the maximum as the incident linear polarization is either along the *c-*axis ($\theta =0^\circ$) or *b-*axis ($\theta =90^\circ$). Also ${I}_{\parallel }\left({B}_{\mathrm{g}}\right)$ will display a four-fold rotational symmetry with the incident polarization angle, where the intensity will be the minimum when the incident linear polarization is along any of the crystal axes. Figure 3c-h display the polar plots of the Raman intensity variations of the parallel polarization components for the Raman modes at 83, 129, 167, 276, 342, and 454 cm^−1^ as a function of the incident polarization angle. The Raman mode at 83 cm^−1^ is a B_g_ mode as it shows a four-fold rotational symmetry, while all the other modes are A_g_ modes giving a two-fold rotational symmetry. The A_g_ modes at 167, 276, 342, and 454 cm^−1^ attain a maximum intensity when the incident polarization is along the *x-*direction ($\theta =0^\circ$), while the A_g_ mode at 129 cm^−1^ gets a maximum intensity when the incident light is polarized along the *y-*direction ($\theta =90^\circ$). Therefore, it can be inferred that the direction of rippling which corresponds to the *c-*axis of the crystal is oriented along either the *x-*direction or the *y-*direction.

### Linear dichroism in cannizzarite crystal

**Figure 4:**
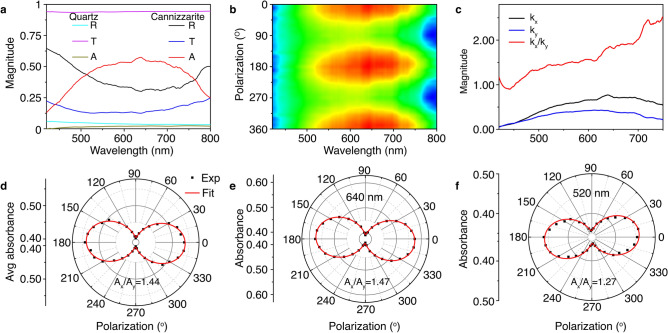
(**a**) Measured reflectance (*R*), transmittance (*T*), and absorbance (*A*) spectra from the 92 nm-thick cannizzarite flake. The cyan, magenta, and brown curves are the measured *R*, *T*, and *A* from the bare quartz substrate. (**b**) Contour color map showing the evolution of the absorbance spectra as a function of the incident polarization angle. (**c**) Imaginary part of the refractive index as a function of the wavelength for incident linear polarizations along the *x*-direction (*k*_x_) and the *y*-direction (*k*_y_), together with the *k*_x_/*k*_y_ ratio indicating the linear dichroism in cannizzarite crystal. (**d**) Polar plot of the evolution of the average absorbance over the measured wavelength range. (**e,f**) Polar plots of the evolution of the absorbance at the wavelengths of 640 nm and 520 nm.

Next, the effect of structural anisotropy on the anisotropic optical properties of cannizzarite crystal is explored by performing polarization-dependent absorption measurements in the 92 nm-thick flake. Figure 4a plots the measured reflectance (*R*), transmittance (*T*), and absorbance (*A*) spectra in the 420–800 nm wavelength range when the incident white light is linearly polarized along the *x*-direction at *θ* = 0°. The reflectance spectrum (black curve) shows a broad dip around 650 nm, while the transmittance spectrum (blue curve) also shows a shallow dip around 550 nm. The absorbance spectrum of the cannizzarite flake on the quartz substrate is then obtained using the relationship *A* = 1 − *R* − *T*. The effect of absorption in the quartz substrate is measured by obtaining *R* (cyan curve), *T* (magenta curve), and *A* (brown curve) from the bare quartz substrate. Finally, the absorbance spectrum of the cannizzarite flake (red curve) is obtained by subtracting the absorbance of the bare quartz from the absorbance recorded from the cannizzarite flake on the quartz. The absorbance of cannizzarite shows a broad peak centered around 640 nm. The contour color map in Fig. 4b displays a series of absorbance spectra for different incident linear polarization angles varied from $0^\circ$ to $360^\circ$. A periodic variation in absorbance as a function of the incident polarization angle is observed, which directly reflects the structural anisotropy in cannizzarite crystal. Further, the imaginary part of the refractive index of the cannizzarite flake is extracted from the reflectance and transmittance spectra by using the equation

5 $$k=\frac{\lambda }{4\pi d}ln\left\{\frac{2T}{\left[{T}^{2}-{\left(1-R\right)}^{2}\right]+{\left\{{\left[{T}^{2}-{\left(1-R\right)}^{2}\right]}^{2}+4{T}^{2}\right\}}^{1/2}}\right\}$$

where *d* is the thickness of the flake. The estimated $k$ values as a function of the wavelength are plotted in Fig. 4c when the incident white light is linearly polarized along the $x$-direction (${k}_{x}$) and the $y$-direction (${k}_{y}$), together with the ${k}_{x}/{k}_{y}$ ratio representing the quantification of linear dichroism. It is observed that the ${k}_{x}/{k}_{y}$ ratio is greater than 1 in the entire wavelength range, while its value gradually increases from approximately 1 at 420 nm to 2.52 at 750 nm, suggesting that cannizzarite crystal exhibits strong linear dichroism. It should be noted that for the estimation of the *k* value of cannizzarite we have not included the effect of multiple reflections of light within the cannizzarite flake at the cannizzarite-quartz and cannizzarite-air interfaces. Therefore, the estimated *k* value would be slightly different from the actual *k* value in the crystal. Nevertheless, the current estimated ${k}_{x}/{k}_{y}$ ratio clearly shows that cannizzarite crystal has strong linear dichroism effect. The polar plot in Fig. 4d shows the evolution of the average absorbance integrated over the measured wavelength range as a function of incident polarization angle. The two-fold rotational symmetry in the evolution of the absorbance is fitted with a periodic function of the form $A={A}_{x}{\cos}^{2}\theta +{A}_{y}{\sin}^{2}\theta$, where ${A}_{x}$ and ${A}_{y}$ are the absorbance values when the incident polarization is along the *x-*direction and the *y-*direction, respectively. The absorbance reaches the highest value as the incident linear polarization is along the *x*-direction. Previously, other natural vdW heterostructure materials such as cylindrite^32^, franckeite^37^ and lengenbachite^35^ have showed the similar characteristics with the absorption being maximum when the incident linear polarization is along the rippling direction of the *c-*axis of the crystal. Therefore, it can be concluded that the *c-*axis along the direction of ripples of the cannizzarite crystal is oriented along the *x-*direction. Simultaneously, the absorbance exhibits the lowest value when the incident linear polarization is along the *y*-direction, which follows the *b-*axis of the crystal. The average ${A}_{x}/{A}_{y}$ ratio within the wavelength region is 1.44. Figure 4e and f show the polar plots of the evolution of the absorbance at two particular wavelengths of 640 nm and 520 nm, with the ${A}_{x}/{A}_{y}$ ratio of 1.47 and 1.27, respectively.

### Polarization-dependent anisotropic THG from cannizzarite flakes

**Figure 5:**
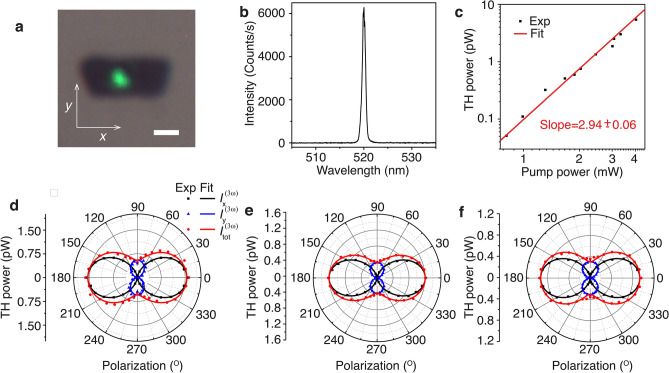
(**a**) Transmission optical microscope image of THG emission from the 92 nm-thick cannizzarite flake when excited with a pump laser beam at 1560 nm. Scale bar is 2 μm. (**b**) Measured THG spectrum showing a peak wavelength at 520 nm. (**c**) Double log-scale plot of the measured TH power as a function of the pump power. (**d**–**f**) Angular dependence of the TH power as a function of the incident linear polarization angle of the pump beam for the cannizzarite flakes with thicknesses of 92, 127, and 173 nm, respectively.

The in-plane structural anisotropy of the crystal also induces anisotropic nonlinear optical properties, as reported in other natural vdW heterostructures of cylindrite^32^, franckeite^36^, and lengenbachite^35^. With that hindsight, we investigate the polarization-dependent anisotropic THG response from the mechanically exfoliated cannizzarite flakes. Figure 5a shows the transmission optical microscope image of the THG emission from the 92 nm-thick cannizzarite flake shown Fig. 1c. The flake is illuminated by a 1560 nm fundamental laser with the spot size of 1.5 µm. The recorded THG spectrum in Fig. 5b shows that the spectral response peaks at 520 nm, which is exactly one-third of the excitation wavelength. The log-scale plot of the average THG power as a function of the average pump power in Fig. 5c shows a cubic power-law dependence confirming the THG process. For the average pump power of 4 mW corresponding to a peak pump irradiance of 27.2 GW cm^−2^, the achieved THG conversion efficiency for the 92 nm-thick flake is around 1.34 × 10^−9^. When the crystal axes $c$, $b$, and $a$ are oriented along the $x$, $y$, and $z$-directions, the contracted form of the third-order susceptibility tensor of the monoclinic crystal system of cannizzarite can be written as^59^,

6 $${\chi }^{\left(3\right)}=\left[\begin{array}{cc}\begin{array}{ccc}\begin{array}{ccc}{\chi }_{11}& 0& 0\\ 0& {\chi }_{22}& 0\\ 0& 0& {\chi }_{33}\end{array}& \begin{array}{ccc}0& 0& {\chi }_{16}\\ {\chi }_{24}& 0& 0\\ 0& {\chi }_{35}& 0\end{array}& \begin{array}{ccc}0& {\chi }_{18}& 0\\ 0& 0& {\chi }_{29}\\ {\chi }_{37}& 0& 0\end{array}\end{array}& \begin{array}{c}0\\ 0\\ 0\end{array}\end{array}\right]$$

where the first subscript 1, 2, 3 refers to *x*, *y*, *z* respectively, and the second subscript signifies the following components

$$\begin{array}{cc}\begin{array}{ccc}\begin{array}{ccc}xxx& yyy& zzz\\ 1& 2& 3\end{array}& \begin{array}{ccc}yzz& yyz& xzz\\ 4& 5& 6\end{array}& \begin{array}{ccc}xxz& xyy& xxy\\ 7& 8& 9\end{array}\end{array}& \begin{array}{c}xyz\\ 0\end{array}\end{array}$$

The fundamental beam linearly polarized at an angle $\theta$ with respect to the *x-*axis at frequency $\omega$ can be expressed as ${\overrightarrow{E}}^{\left(\omega \right)}={E}_{0}\left(\cos\theta \widehat{x}+\sin\theta \widehat{y}\right)$, with $\widehat{x}$ and $\widehat{y}$ being the unit vectors along the *x-* and *y*-axes. Since the polarization of the excitation electric field only remains in the *x*–*y* plane, the contribution from the χ^(3)^ components including *z* terms to the THG emission is negligible. Therefore, the *x-* and *y-*polarized components of the THG intensity can be written as,

$${I}_{x}^{\left(3\omega \right)}\propto {\left({\chi }_{11}{\cos}^{3}\theta +3{\chi }_{18}\cos\theta {\sin}^{2}\theta \right)}^{2}$$

7 $${I}_{y}^{\left(3\omega \right)}\propto {\left({\chi }_{22}{\sin}^{3}\theta +3{\chi }_{29}\sin\theta {\cos}^{2}\theta \right)}^{2}$$

Figure 5d plots the evolution of the TH power as a function of the incident linear polarization angle with respect to the *x-*axis at the pump power of 2.5 mW. The desired incident polarization is obtained by placing a linear polarizer oriented along the *x*-axis (the $c$-axis of the crystal) and a rotating half-wave plate. The measured *x-* and *y-*polarized components (black and blue data points) of the TH power are theoretical fitted (solid curves) using Eq. (7), showing a good agreement between the theoretical prediction and the measurements. The red data point and the curve correspond to the total TH power. The polarization-dependent THG response from the cannizzarite flake displays an anisotropic two-fold rotational symmetry, with the maximum THG emission occurring at the incident linear polarization angle of $\theta =0^\circ$, which is parallel to the rippling direction along the $c$-axis of the crystal. Similar trends are also observed in other flakes with thicknesses of 127 and 173 nm, as shown in Fig. 5e and f. As the incident linear polarization is along the *c-*axis of the crystal, the measured THG conversion efficiency from the three flakes with the thicknesses of 92, 127, and 173 nm are 6.01 × 10^−10^, 4.80 × 10^−10^, and 3.52 × 10^−10^ respectively, at the pump power of 2.5 mW with the incident spot size of 1.5 $\mu$m, which corresponds to a pump power irradiance of 17 GW cm^−2^. The THG anisotropy ratio ${I}_{tot }^{\left(3\omega \right)}\left(\theta =0^\circ \right)/{I}_{tot }^{\left(3\omega \right)}\left(\theta =90^\circ \right)$ almost maintains a constant value of 3.24 ± 0.14 for all the three flakes with different thicknesses. The anisotropy in the THG response is further revealed by extracting the average relative magnitudes of ${\chi }^{\left(3\right)}$ tensor elements for the cannizzarite flakes with different thicknesses as ${\chi }_{11}:{\chi }_{18}:{\chi }_{22}:{\chi }_{29}=1:0.34:0.57:0.22$.

Finally, to compare the THG response of cannizzarite with other existing nonlinear 2D materials, we estimate the effective scalar value of the third-order nonlinear susceptibility ${\chi }_{eff}^{(3)}$ by using the following expression

8 $${\chi }_{eff}^{(3)}={\left(\frac{16\sqrt{{n}_{3}^{2}+{k}_{3}^{2}}{n}_{1}^{3}{\epsilon }_{0}^{2}{c}^{4}{f}_{rep}^{2}{W}^{4}{\tau }^{2}{\left[\frac{\pi }{4\ln 2}\right]}^{3}{P}^{\left(3\omega \right)}}{9{\omega }^{2}{d}^{2}{{P}^{\left(\omega \right)}}^{3}} \left(\frac{\left(\frac{4{\pi }^{2}{k}_{3}^{2}{d}^{2}}{{\lambda }_{3}^{2}}\right)}{{e}^{-\frac{4\pi {k}_{3}d}{{\lambda }_{3}}}-2{e}^{-\frac{2\pi {k}_{3}d}{{\lambda }_{3}}}+1}\right){e}^{\frac{4\pi {k}_{3}d}{{\lambda }_{3}}}\right)}^\frac{1}{2}$$

where ${\epsilon }_{0}$ is the vacuum permittivity, $d$ is the flake thickness, ${P}^{\left(3\omega \right)}$ represents the average TH power, ${n}_{1}$ and ${n}_{3}$ are the real part of the refractive index of cannizarite at the fundamental wavelength ${\lambda }_{1}=1560$ nm and the TH wavelength ${\lambda }_{3}=520$ nm, while ${k}_{3}$ is the imaginary part of the refractive index at ${\lambda }_{3}$. The parameters of the fundamental beam are given by spot size $W$ = 1.5 μm, repetition rate ${f}_{rep}$= 80 MHz, pulse width $\tau$ = 90 fs, while ${P}^{\left(\omega \right)}$ is the pump power. The value of ${k}_{3}$ is estimated to be 0.48 from the absorbance measurement in Fig. 4. It is noted that the refractive index of cannizzarite crystal is unknown, but the refractive indices of its component binary sulfides are available, with ${n}_{1}=$ 4.24 and ${n}_{3}=$ 4.34 for PbS, ${n}_{1}=$ 1.9 and ${n}_{3}=$ 2.6 for Bi_2_S_3_^60^. By assuming an averaged refractive index of ${n}_{1}={n}_{3}=$ 3.5 for cannizzarite crystal, the estimated ${\chi }_{eff}^{(3)}$ values for the three flakes with the thicknesses of 92, 127, 173 nm are found to be $1.61\times {10}^{-20}, 1.42\times {10}^{-20}$, and $1.41\times {10}^{-20}$ m^2^ V^−2^ respectively, which are quite close to each other. Although the third-order nonlinear susceptibility value of cannizzarite is less compared to other anisotropic natural vdW heterostructures such as cylindrite ($3.06\times {10}^{-19}$ m^2^ V^−2^)^32^, franckeite ($1.87\times {10}^{-19}$ m^2^ V^−2^)^36^, and lengenbachite ($2.18\times {10}^{-19}$ m^2^ V^−2^)^35^, the THG anisotropy ratio in cannizzarite (3.24) is much higher than those in cylindrite (2.25), franckeite (2.60), and lengenbachite (1.90).

## Discussion

In summary, we have demonstrated that ultrathin layers of vdW heterostructures composed of alternating PbS-type and Bi_2_S_3_-type 2D material layers can be obtained by the mechanical exfoliation of natural layered mineral cannizzarite. Compared to other natural vdW heterostructure materials such as cylindrite, franckeite and lengenbachite, it is relatively difficult to exfoliate cannizzarite crystal into ultrathin flakes. The smallest thickness achieved in this study by mechanical exfoliation is 92 nm, corresponding to 59 pairs of layers of the vdW heterostructure. A better controlled mechanical exfoliation or other exfoliation approaches such as liquid phase exfoliation could be utilized to produce 2D vdW heterostructures of molecular thicknesses. Through the TEM analysis, we have established that the forced commensuration between the constituent lattices in cannizzarite crystal leads to in-plane structural anisotropy. The bulk and surface chemical composition of cannizzarite crystal is further determined by the EDXS and XPS analysis as Pb_59_Bi_67_S_97_Se_30_ and Pb_47_Bi_56_S_107_Se_20_, respectively. It is demonstrated that the structural anisotropy in thin cannizzarite flakes can be optically probed by angle-resolved polarized Raman spectroscopy, which is used to identify the direction of the ripples along the *c*-axis of the crystal. By performing the polarization-dependent absorption measurements, it is found that cannizzarite crystal exhibits strong linear dichroism, which might be useful for designing polarization-sensitive photodetectors and modulators. Furthermore, the effect of structural anisotropy on the nonlinear optical properties of cannizzarite is studied by demonstrating anisotropic THG response from thin cannizzarite flakes. The TH power for the incident polarization along the rippling direction of the crystal is found to be almost 3.24 times stronger compared to that along the direction perpendicular to it. These results provide deeper understanding of the origin of optical anisotropy in the natural vdW heterostructure of cannizzarite, even though its constituent 2D material layers are structurally isotropic in nature. The third-order nonlinear susceptibility of cannizzarite is measured as 1.61 $\times {10}^{-20}$ m^2^ V^−2^, indicating that cannizzarite exhibits strong THG response as other existing nonlinear anisotropic vdW materials. The study presented here may be harnessed for building polarization-sensitive anisotropic linear and nonlinear photonic devices used in future on-chip optical computing, optical information processing and communication.

## Methods

### Sample preparation

The quartz substrate is sonicated in deionized water, acetone, and isopropanol one after the other. The cannizzarite thin flakes are mechanically exfoliated from the bulk natural cannizzarite mineral (from La Fossa Crater, Vulcano Island, Lipari, Eolie Islands, Messina Province, Sicily, Italy) using Nitto tape (SPV 224) and transferred directly onto the cleaned quartz substrate. For preparing the TEM sample, the exfoliated thin flakes are first transferred onto a thermal release tape (Revalpha 319Y-4LS). Then the flakes from the thermal release tape are transferred on to a copper TEM grid by placing the tape on it and heating at $100^\circ{\rm C}$ for 30 s.

### TEM and EDXS measurement

The TEM imaging and EDXS measurements of the transferred cannizzarite flakes on a copper TEM grid are performed using a JEOL 2100 scanning/transmission electron microscope (S/TEM) operating at 200 keV, with a Bruker Quantax X-Flash detector for EDXS compositional analysis.

### XPS measurement

The XPS spectra from the exfoliated cannizzarite flakes are obtained using the Thermo Scientific Nexsa XPS spectroscopy system. The instrument is equipped with a monochromatic Al Kα X-ray source. The highest energy resolution of the recorded XPS spectra are < 0.48 eV. Small spot spectroscopy is achieved by inserting an aperture into the electrostatic lens column forming a virtual probe at the surface.

### Optical setup

The Raman spectrum is acquired by using a 632.8 nm He–Ne laser beam, which is focused on the cannizzarite flake using a 40 × objective lens (NA = 0.6). The linear polarization of the laser beam is determined using a linear polarizer and a rotating half-wave plate. The back-reflected light is further collected by the same objective lens and guided towards a spectrometer (Horiba, iHR 550) using a beam splitter. The Rayleigh-scattered laser light is filtered out by placing a long pass filter (Semrock, LP02-633RE-25) in front of the spectrometer. The parallel and perpendicular polarization components of the Raman spectrum are analyzed using another linear polarizer before the spectrometer.

For the polarization‐dependent absorption measurements, white light beam from a broadband white light source (Thorlabs, SLS201L, 360–2600 nm) is passed through a linear polarizer and focused on the cannizzarite flake using a 100 × objective lens (NA = 0.7). The reflection spectrum is analyzed from the back‐reflected light from the sample which is collected by the same objective lens and directed towards the spectrometer with a beam splitter. The transmission spectrum is collected using another 100 × objective lens (NA = 0.7). An iris is used to spatially filter out a small area of the flake in both the cases. The reflectance (*R*) and transmittance (*T*) are measured by normalizing the reflection and transmission spectra with the light source spectra. Finally, the absorbance (*A*) spectrum is obtained by using the relationship *A* = 1 − *R* − *T*.

The THG measurements are performed by designing a nonlinear transmission microscopy system. The pump laser beam at the fundamental wavelength of 1560 nm (Calmar fiber laser, pulse width 90 fs, repetition rate 80 MHz) is focused on the cannizzarite flake using a 40 × objective lens (NA = 0.65). The excitation linear polarization of the pump laser is determined by using a linear polarizer and a half-wave plate in front of the objective lens. The transmitted THG emission from the flake is collected by a 100 × objective lens (NA = 0.7) and analyzed using a spectrometer (Horiba, iHR 550). A short pass filter (Thorlabs, FESH 900) is used before the spectrometer to remove the transmitted fundamental pump laser beam. The polarization state of the collected THG spectrum is resolved by using another linear polarizer before the spectrometer.

## Data Availability

The datasets generated during and/or analysed during the current study are available from the corresponding author on reasonable request.
